# JNK and p38 gene and protein expression during liver ischemia-reperfusion in a rat model treated with silibinin

**DOI:** 10.22038/IJBMS.2022.60550.13422

**Published:** 2022-11

**Authors:** Nastaran Aamani, Abouzar Bagheri, Neda Masoumi Qajari, Majid Malekzadeh Shafaroudi, Abbas Khonakdar-Tarsi

**Affiliations:** 1 Department of Clinical Biochemistry and Genetics, Faculty of Medicine, Mazandaran University of Medical Sciences, Sari, Iran; 2 Genetic Biology, Islamic Azad University of Tonekabon, Tonekabon, Iran; 3 Department of Anatomy and Biology, Faculty of Medicine, Mazandaran University of Medical Sciences, Sari, Iran; 4 Faculty of Medicine, Immunogenetic Research Center (IRC), Mazandaran University of Medical Sciences, Sari, Iran

**Keywords:** Ischemia, JNK, p38, Reperfusion, Silibinin

## Abstract

**Objective(s):**

Signal transduction of mitogen-activated protein kinases (MAPKs) is activated during ischemia. In this study, c-Jun N-terminal Kinase (JNK) and p38 MAPK (p38) gene and protein expression were evaluated as two members of the MAPK family during liver ischemia-reperfusion in rats.

**Materials and Methods:**

Thirty-two male Wistar rats were divided into four groups of eight: Vehicle, ischemia-reperfusion (IR), ischemia-reperfusion+silibinin (IR+SILI), and SILI. The IR and IR+SILI groups differed from the other two groups in that they underwent one hour of ischemia followed by three hr of reperfusion. The Vehicle and IR groups received normal saline while the SILI and IR+SILI groups were treated with silibinin (50 mg/kg). At the end of the reperfusion time, blood and ischemic liver tissue were collected for further experiments.

**Results:**

The expression of JNK and p38 gene, the amount of serum hepatic injury indices, and malondialdehyde (MDA) in the IR group increased significantly compared with the vehicle group. The JNK and p38 gene expression decreased significantly in the IR + SILI group compared with the IR group. Glutathione peroxidase (GPx) and total antioxidant capacity (TAC) levels decreased in the IR group while increasing in the IR+SILI group. Histological examination showed that silibinin significantly reduced the severity of hepatocyte degradation. Western blot results were completely consistent with real-time PCR results.

**Conclusion:**

The possible pathways of the protective effect of silibinin against hepatic ischemia damages is to reduce the expression of the p38 and JNK gene and protein.

## Introduction

Intact blood supply is vital for necessary oxygen and nutrient transport to different tissues. There are various occasions on which ischemia and restriction in blood supply to tissue can appear, including trauma, surgery, infection, transplantation, and tumors. The duration of ischemia and the kind of organs could lead to different results of ischemic damage. Liver tissue is one of the most ischemia-sensitive tissues because its exclusive blood supply is mainly from portal venous perfusion (1). 

Liver disorders are among the main reasons for human death. Liver transplantation, surgery, or resections are the choice solutions for patient treatment. Several studies proved that complications of ischemia-reperfusion (IR) are the main reasons for unsuccessful liver transplantation surgery. Liver IR often leads to acute inflammatory responses and increases oxygen radicals and inflammatory cytokines. Accordingly, it can be associated with other molecular mechanisms such as necrosis, apoptosis, and autophagy, where tissue damages are more likely to occur under such a chain of reaction. Besides, it can be expected that gene expression, cell signaling, mutation, and cell death (apoptosis) might change after IR. It is because the effects of ischemia may develop into vital biomolecules like nucleic acids after reperfusion injury (2). 

Many apoptosis signals like mitogen-activated protein kinase (MAPK) families, which include three subfamilies called p38 mitogen-activated protein kinases, c-Jun N-terminal kinase (JNK), and extracellular signal-regulated kinases (ERKs), are triggered by reactive oxygen and reactive nitrogen species (RNS, ROS). p38 and JNKs are also well-known as the stress-activated protein kinase (SAPK) (3). The natural process of the pathways of ERK and p38 is essential to produce tumor necrosis factor-alpha (TNF-α), interleukin 6 (IL-6), IL-1, IL-8, and JNKs to generate nuclear factor kappa B (NF-κB), activator protein 1 (AP1), and hypoxia-inducible factor 1 (HIF-1) as well (4). So, p38 and JNKs are activated under oxidative stress conditions and are possibly related to cellular damage in the IR point.

Numerous natural antioxidants have been extracted and applied to neutralize the adverse effects of cellular oxidative stress and minimize IR injuries (IRI). Silymarin, a liver-protective extract of *Silybum marianum*, is mainly composed of silibinin as the main effective ingredient (5). Silibinin plays a radical scavenger and neutralizer role in oxidative stress conditions like IR. Therefore, considering the relationship between JNK & p38 signaling pathways and oxidative stress, the study focused on the silibinin impacts on the JNK and p38 gene expression during warm hepatic IR.

## Materials and Methods

***Experimental Procedures***

Thirty-two male Wistar rats weighing 220 to 250 g were entered into the study. The rats were maintained in a uniform environment whose temperature was set to be 22±2 ^°^C, with an approximate humidity of 55±5%, and a 12/12 hr light/dark cycle. The animals received a standard diet containing desirable amounts of protein, fat, and carbohydrates. Eighteen hr before surgery, nutriment was removed from the animal cage, but the water was freely accessible. The rats were randomly divided into four groups of eight: 

1. In the control (Vehicle) group, normal saline was intraperitoneally (IP) injected twice (30 min before laparotomy and immediately after returning the liver into the body). 

2. In the IR group, normal saline was injected twice similar to the control group, but with the difference that 1 hr ischemia and 3 hr reperfusion were also applied. 

3. The rats in the SILI group were operated on the same as the Vehicle group (without ischemia) and received silibinin (lyophilized powder with a dihydrogen succinate disodium salt formula (Legalon ^®^ SIL) manufactured by Rottapharm/Madaus (Cologne, Germany), dissolved in 0.9% sodium chloride solution, 50 mg/kg twice (30 min before ischemia and immediately after reperfusion) instead of saline. 

4. In the IR+SILI, the rats were insulted by IR like the IR group but received silibinin two times (6). 

The study was conducted according to the guidelines of the Declaration of Helsinki and approved by the Ethics Committee of Mazandaran University of Medical Sciences (IR.MAZUMS.REC.1397.288 and 2018-06-27).

***Surgery***

To achieve similar conditions, all surgical operations were carried out between 8 and 13 AM. Ketamine 60 mg/kg and Xylazine 8 mg/kg with IP injection were used to anesthetize the rats. The liver was removed from the body of both Vehicle and SILI groups after laparotomy and was then entered into the body after 1 hr without any handling. For the other two groups (IR and IR+SILI), the triad of the hepatic artery, portal vein, and bile duct from the middle and left hepatic lobes were blocked by a clamp. Blood samples of the lower Humphrey vein were gathered from all four groups after 3 hr of reperfusion, and the separated serum was stored at 20 ^°^C to ascertain biochemical markers. To determine the expression of JNKs and p38 genes, dissociated tissue samples were loaded into RNAlater (Qiagen). Part of the tissue was also fixed in formalin for histology (6). 

***Real-time RT-PCR***

*RNA isolation*

As a method to evaluate the expression of JNK and p38 genes, a Qiagen company kit was used to extract total RNA from the liver tissue according to the manufacturer’s protocol. The concentration of total RNA was determined by using a NanoDrop spectrophotometer, based on absorbance at 260 nm. The absorbance ratio of 260/280 nm was utilized to survey the purity of the RNA which is almost 2 for RNA purity. Isolated RNA (1.5 μl) was loaded into the NanoDrop, its absorption was estimated at 260 nm and in the ratio of 260/280.

***RNA integrity analysis***

Electrophoresis [1.5 % agarose gel (Agarose type I (LE), Sigma, USA)] was performed to evaluate the quality and integrity of the extracted RNA. Bonds were visible via the UV-doc device and photos were taken through a transilluminator upon completion of the electrophoretic separation. There is a link between the quality of bond images and the quantity of RNA (a 28S:18S rRNA ratio of 2:1).

***Synthesis of cDNA***

Five micrograms of total RNA were used according to the protocol of the NOYA kit. Initially, solutions were incubated in the thermal cycler for 10 min at 25 ^°^C, followed by 60 min at 42 ^°^C and 3 min at 95 ^°^C. Before applying the real-time PCR process, the synthesized DNA was kept at -20 ^°^C. 

***Real-time PCR***

Finally, real-time PCR was accomplished in duplicate form and used GAPDH as the control gene to assess the JNK and p38 gene expression. The PCR cycles and the sequences of primers are shown in Tables 1 and 2, respectively.

To distinguish the yield of specific PCR products, green light with a 470 nm wavelength was flashed at samples, and the fluorescence signals were measured through 510 nm. The trace fluorescence signals were found at 72 ^°^C, which means it can minimize the casualties of primer dimers. Eventually, melt curve analysis was used at temperature ranges from 70 ^°^C to 95 ^°^C, and a specific bond was used to ensure the accuracy and reliability of the real-time PCR reaction.

***Assessment of PCR specificity and efficiency***

The PCR products were fixed in agarose gel 2 % containing SYBR Green with 85 V for 40 min. Also, the standard curve was drawn to determine PCR efficiency.

***Serum enzymes activity***

Serum was separated from the collected blood samples through centrifuging at 3000 rpm for 5 min. The serum activity of ALT, AST, ALP, and LDH were determined by applying the Pars Azmoon kit and Hitachi biochemical autoanalyzer.

***Total antioxidant capacity (TAC), malondialdehyde (MDA) level, and glutathione peroxidase (GPx) activity in liver tissue***

TAC was measured by ZellBio kit using a colorimetric method and following the reduction of Fe^3+^ to Fe^2+^ by antioxidant compounds at a wavelength of 460–490 nm. Subsequently, TAC concentration was assessed based on the standard chart to account for the ratio of the millimolar per sample. The formula was: ΔOD=OD2-OD1

The MDA measurements were taken with a ZellBio kit using a colorimetric method based on thiobarbituric acid reactive substances (TBARSs). The absorptions of samples were taken by the spectrophotometer at 535 nm. MDA data were then calculated using the standard curve.

GPx activity was measured at 412 nm employing the colorimetric method (ZellBio kit). GPx activity was calculated based on the following formula:

GPx _activity_ (U/ml)=(OD _control_-OD _sample_) / (OD _standard_-OD_blank_)×6000

***Histopathological examination***

Liver tissue samples***,*** a length of 2-3 mm, were fixed with neutral formalin (10 % formaldehyde in PBS) for 18-24 hr. Afterward, samples were prepared to embed in paraffin in some steps. Having poured molten paraffin in L1 blocks along with the tissue depth, the current researchers sectioned off the tissue by a microtome at a thickness of 2-8 μm. The sections containing paraffin were placed on the slide using Mayer’s albumin solution and were kept at a temperature of 60 ^°^C for two hours. Deparaffinization and dehydration were carried out, and slides were stained with hematoxylin and eosin. Eventually, the stained slides were explored and shot by an optical microscope.

***Western blot analysis***

Total liver tissue protein of different groups was extracted using cold lysing buffer containing phenylmethylsulfonyl fluoride (1 mM) according to the instructions of the manufacturer. Equivalent amounts of proteins were denatured by boiling in sample buffer (reducing conditions) for 5 min and then analyzed on 12.5% ​​sodium dodecyl sulfate-polyacrylamide gel electrophoresis (SDS-PAGE). Proteins were transferred to a pre-activated polyvinylidene fluoride (PVDF) membrane overnight. The gel was stained with Coomassie brilliant blue to confirm that the proteins were completely transferred. Nonspecific binding was blocked with 5% nonfat milk in Tris-buffered saline (TBS) containing 0.05% Tween-20 (TBST) for 2 hr at room temperature. P-p38, P-JNK, p38, and JNK were detected by incubation with rabbit anti-rat primary antibodies (1:500, Bioworld Technology, USA) at 4 ^°^C overnight. The membrane was then incubated with a secondary antibody (goat anti-rabbit IgG conjugated with horseradish peroxidase, 1:2000; Bioworld Technology, USA) for 2 hr at room temperature. The membrane was washed three times in TBST and was exposed to a sufficient amount of luminol solution (Santa Cruz Carlsbad, CA, USA) for 1 to 3 min.

***Data analysis***

The findings were shown as mean±standard deviation of the mean (mean±SD). Employing the SPSS 18 software package, the mean difference among groups was estimated by one-way ANOVA and Tukey’s multiple comparison tests using *P*<0.05 as the level of significance. Real-time PCR results were analyzed considering the following formula:

RQ = 2^−ΔΔCt^, where ΔΔCt=ΔCt (test)–ΔCt (calibrator) (7).

## Results

***Gene expression***

**Figure 1.**
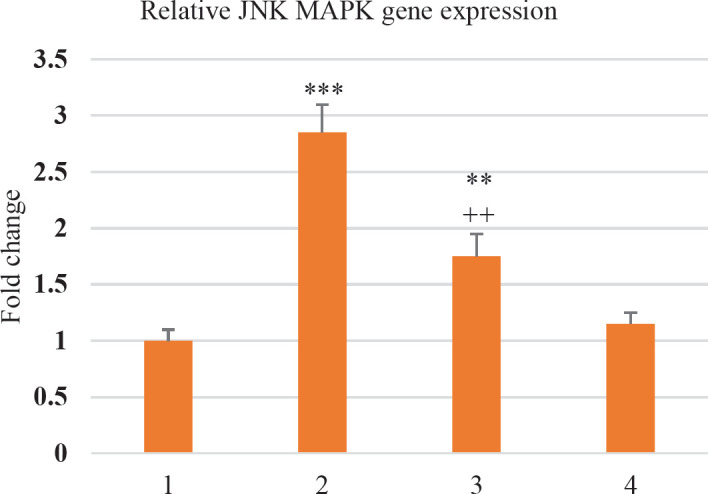
Changes in the JNK gene expression in liver tissue of four studied groups

**Figure 2:**
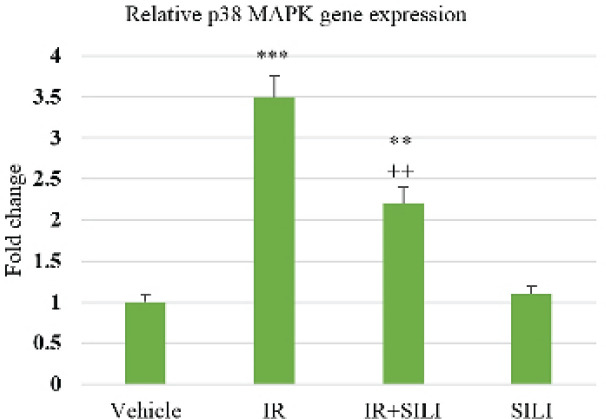
Variation of p38 gene expression during liver IR under the influence of silibinin

In the analysis of the mRNA expression, there was a remarkable difference between the IR and Vehicle groups. In comparison with the Vehicle group, the expression of both JNK and p38 genes was significantly elevated in the IR group. The IR+SILI group witnessed a noticeable decline in the two genes’ expression by silibinin treatment compared with the IR group. There were no considerable differences between SILI and the Vehicle groups (Figures 1 and 2).

***Serum and tissue indicators***

A great deal of intracellular enzymes, aspartate transaminase (AST), alanine transaminase (ALT), and lactate dehydrogenase (LDH), were entered into the serum under hepatic IR conditions. Whereas the amount of MDA increased in the tissue, that of TAC and GPx decreased. Besides, while the silibinin injection caused a remarkable decline in serum LDH, ALT, AST, and MDA, the liver witnessed an increase in the level of TAC and GPx. However, there were no valuable changes in the amount of serum alkaline phosphatase (ALP) (Table 3).

***Histological results***

**Figure 3:**
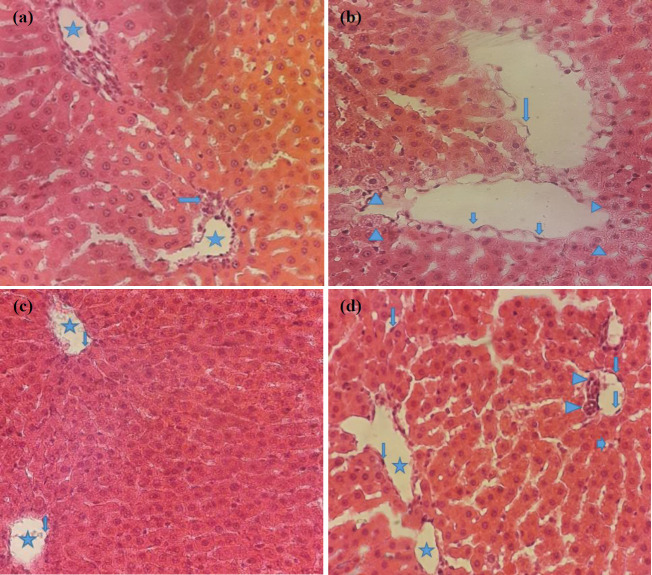
Liver X400 magnification in H&E staining

Vehicle group (Figure 3A): The tissue of hepatic classical lobules, including Remake’s hepatocyte cords, lobular central veins, portal spaces, and sinusoids seem normal. The central vein and its endothelial cells are viewed as salutary and intact. In zone I of the hepatic classical lobule, a bile ductule with a simple cubic epithelium in the duct wall and a transverse longitudinal section of the hepatic artery branch are visible in the margin of the classic lobule and inside the portal space. The endothelium of the branch of the hepatic artery with prominent nuclei into the lumen indicates arterial health. Hepatocytes are visible with clear boundaries and a nucleus with clear nuclei. 

IR group (Figure 3B): Many hepatocytes in the area of one and two classic hepatic lobules around the portal space, containing the bile duct and portal vein branching, show the normal density of mitochondria. However, with the emergence of different size apoptotic vacuoles and to varying degrees, with less pale chromatin in the nucleus and less cytoplasmic staining, the fusion process, and destruction of mitochondria in the area of the three classic hepatic lobules were observed following ischemia. Although endothelial cells of the sinusoidal wall have desquamated in some areas, the endothelium lining the central lobular veins is still intact, and they have ruptured from beneath their thin connective tissue. Decreased staining of hepatocytes cytoplasm in zone 3 indicates mitochondrial degradation. Lowered staining of hepatocyte nuclei can be caused by the destruction of part of the DNA.

IR+SILI group (Figure 3C): The hepatic tissue structure, including hepatic artery and portal vein branches in portal space, is undamaged. Besides, the epithelial structure of the bile ductule wall and zones one, two, and three of the classical liver lobules up to the central vein appear intact. In general, negligible damage to liver tissue is observed compared with the IR insulted liver.

SILI group (Figure 3D): Portal space and its components, including the blood vessel branches and the bile ductules, look healthy and untouched. High-pigmented hepatic cords with healthy and clear nuclei and minimal apoptotic vacuoles show the health of the liver tissue in the portal space and the area around it. Unchanged sinusoidal spaces with a covering of intact endothelium and numerous Kupffer macrophage cells are visible. In general, the presence of the protective substance silibinin did not cause any damage to the hepatic tissue structure.

***Western blotting***

**Figure 4:**
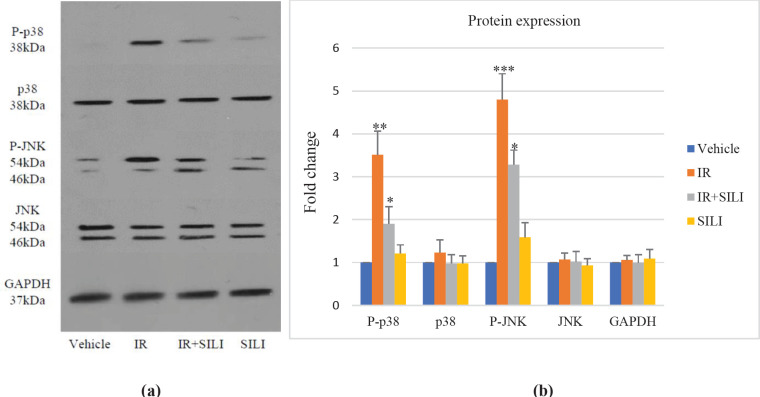
Western blotting analysis for phosphorylated and total JNK and p38 was performed

Western blotting was performed to determine the amount of JNK and p38 proteins in each of the four groups (Figure 4a). The results were consistent with the results obtained from real-time PCR. The amount of JNK and p38 phosphorylated protein in the IR group showed a significant increase compared with the control group (Figure 4B). The amounts of JNK and p38 phosphorylated protein in the SILI+IR group were considerably reduced compared with the IR group, showing positive effect of silibinin on hepatocytes. The amount of protein in the Vehicle and SILI groups was not significantly different.

**Table 1:** Real-time PCR stages and conditions for JNK and p38 gene amlification

**Stage**	**Temperature (°C)**	**Time**	**Cycle**
Initial denaturation	95	12 min	1
Denaturation	95	15 sec	40
Annealing	JNK: 60 p38: 60 GAPDH: 62	25 sec	
Extension	72	20 sec	
Final extension	72	5 min	1

**Table 2:** Primer sequence and amplicon length for real time reaction of investigated genes

Gene	Primer sequence 5'→3'	Amplicon length
JNK	Sense: 5' - CACCACCAAAGATCCCTGACA -3' Antisense: 5'- GCACCTAAAGGAGACGGCTG -3'	145 bp
p38	Sense: 5' - TCATAGGCATCCGAGACATCC -3' Antisense: 5'- CGTCTCCATGAGGTCCTGAAC -3'	83 bp
GAPDH	Sense : 5'- GAAGGTCGGTGTGAACGGATTTG -3' Antisense: 5'- AATGAAGGGGTCGTTGATGGC -3'	103 bp

Silibinin diminished the increased expression of JNK in the IR+SILI group compared with the IR group. The findings are shown as mean±standard deviation (mean±SD) by eight rats in each group. While the symbols *** and ** show a significant difference compared with the Vehicle group, *P<*0.001 and *P<*0.01, respectively, the symbol ^++^displays a significant difference compared with the IR group (*P<*0.01)IR: Ischemia-reperfusion; IR+SILI: ischemia-reperfusion+silibinin; SILI: silibinin

p38 gene expression was reduced by silibinin in IR+SILI group compared with the IR group. The results are shown as mean±standard deviation (mean±SD) by eight rats in each group. The symbols ^*** ^and ^**^ show a significant difference compared with the Vehicle group, *P<*0.001 and *P<*0.01, respectively, and the symbol ^++^ displays a significant difference compared with the IR group (*P<*0.01)

IR: Ischemia-reperfusion; IR+SILI: ischemia-reperfusion+silibinin; SILI:silibinin

**Table 3:** Mean and standard deviation of seven studied items in blood samples collected from rats

**Group**	**Vehicle (Mean ** **±** **SD)**	**IR ** **(Mean ** **±** **SD)**	**IR + SILI (Mean ** **±** **SD)**	**SILI ** **(Mean ** **±** **SD)**
**Index**				
SGOT (U/L)	232.25±21.6	2256±442.6 ^*^	889.13±152.04 ^+**^	257.25±29.2
SGPT (U/L)	71.89±15.9	1384.7±293.8 ^*^	817.38±324.8 ^+**^	82.51±7.6
MDA (µM)	18.31±1.3	27.81±2.8 ^*^	12.25±1.8 ^+**^	20.37±0.9
**GPx** (U/mL)	109.7±10.8	61.36±22.0 ^***^	82.30±8.5 ^+***^	101.52±5.3
LDH (U/L)	344.12±68.1	2479.5±341.7 ^*^	1506.3±315.0 ^+**^	333.01±26.3
ALP (U/L)	237.25±44.0	238.37±58.0 ^*^	241.0±46.7 ^+**^	227.25±8.2
TAC (m M)	0.189±0.01	0.125±0.02 ^***^	0.136±0.01 ^+***^	0.164±0.02

The symbols^ *^ (*P<*0.01), ^**^ (*P<*0.001), and ^***^ (*P<*0.0001) show a significant difference compared with the Vehicle group. The symbol ^+^ shows a significant difference compared with the IR group (*P<*0.05)

(a) Vehicle group: Central vein and its epithelial cells appear healthy and undamaged. (b) IR group: Emergence of numerous different size apoptotic vacuoles with fading out of nuclear chromatin and lower cytoplasmic staining illustrates fusion and destruction of mitochondria in this area following IR. Although sinusoidal wall endothelial cells desquamated in some districts, the endothelium covering the branches of the central lobular vein still appears healthy. However, endothelial cells were detached from beneath their thin connective tissue. (c) IR+SILI group: Remake’s hepatocyte cords have healthy hepatocytes with clear nuclei and nucleoli. The sinusoidal spaces between them, along with the endothelial cells with dense and elongated nuclei appear unharmed. Central veins (stars) and their endothelium (vertical and blue arrows) look perfectly normal. No tissue damage in liver sections is observed. (d) SILI group: The cholangioles are characterized by a simple cubic epithelium (head of the blue arrow) and central lobular vein (stars). The endothelium lining the central lobular vein is intact (narrow and long vertical arrows). Kupffer cells in sinusoidal space (wide horizontal blue arrows) are well recognizable. Zones one, two, and three of the Remake’s hepatocyte cords and the sinusoidal spaces between them are fully healthy and undamaged

(a, b) Amounts of P-p38 and P-JNK proteins in the Vehicle and SILI groups were not significantly different and were lower compared with the other groups. The expression of P-p38 and P-JNK proteins in the IR group upregulated compared with the Vehicle group. The quantities have significantly decreased in the SILI+IR group. The symbols ^*^ (*P<*0.01) and ^**^ (*P<*0.001) show a significant difference compared with the Vehicle group

## Discussion

In this study, c-Jun N-terminal Kinase (JNK) and p38 MAPK (p38) gene and protein expression were evaluated as two members of the MAPK family during liver ischemia-reperfusion in a rat model. We concluded that the expression of the JNK and p38 gene, the amount of serum hepatic injury indices, and malondialdehyde (MDA) in the IR group increased significantly compared with the vehicle group. JNK and p38 gene expression decreased significantly in the IR+SILI group compared with the IR group. Glutathione peroxidase (GPx) and total antioxidant capacity (TAC) levels decreased in the IR group while increasing in the IR+SILI group.

IR is a traumatic phenomenon that occurs as a result of temporary disruption and restoration of blood flow to the tissue (8). The liver is more susceptible to IR injury than any other organ because just a quarter of its perfusion is provided via the oxygen-rich hepatic artery (9). Hepatic IR occurs in various clinical conditions such as liver biopsy, surgery, and transplantation (10). Factors such as starvation, fatty liver, age, time, severity, and rate of IR happening affect the degree of damage (11). Many primary changes occur in the mitochondrion after anoxia. Obstruction of the respiratory chain in the mitochondrion following oxygen deprivation disrupts the oxidative phosphorylation process, depletes ATP, increases glycolysis and lactic acid, and changes in H^+^, Na^+^, and Ca^2+^ homeostasis (12). In the acute phase of ischemia, Kupffer cells are activated by DAMPs released from damaged hepatocytes. Large amounts of ROS and proinflammatory cytokines, including TNF-α, INF-γ, IL-12, and IL-1 are released followed by activation of the immune response and Kupffer cells. TNF-α and IL-1 increase the synthesis of Mac-1 (a superficial adhesion protein) and IL-8 in neutrophils. IL-1 can also trigger the production of more ROS in neutrophils primarily from the source of NADPH oxidase and increases TNF-α synthesis in Kupffer cells (13). The large volume of ROS is the principal reason for IR damages, causing the peroxidation of membranes, various proteins, and DNA. Consequently, the Kupffer cells and neutrophils exert stimulation effects on each other and intensify the inflammation and tissue lesions (14). The production of proinflammatory cytokines leads to severe inflammatory reactions, necrosis, and apoptosis of hepatocytes (15).

One of the signaling pathways discussed in IR is MAPK, which plays a prominent role in intracellular signal transmission in response to extracellular stimuli (16). Among the mammalian MAPKs, p38 and JNK are stimulated by various cellular stresses such as IR.

There is ample evidence to suggest a role for MAPKs, especially p38 and JNK, in liver IRI (17). p38 and JNK are phosphorylated and activated a few minutes after reperfusion, and their activation is associated with induction of apoptosis and necrosis (18). Massip *et al*. approved that treatment with p38/JNK activators causes an increase in transaminase levels and grade 3 necrosis in rat liver tissue. As a result, it can be declared that the inhibition of MAPKs guides the reduction of hepatic IRI ^24^. Research has explicated that p38 and JNK are involved in liver fibrosis (19). One study showed that the activity of JNK and p38 was higher in active hepatic stellate cells (HSCs) than in inactive ones (20). HSCs are responsible for the synthesis of type 1 collagen after liver injury, and their activation usually causes liver fibrosis. JNK is activated by the TNF-α and IL-1 cytokines, which are the principal mediators of IR-induced liver damage (21).

Inflammatory mediators such as eicosanoids, TNF-α, IL-1, adhesive molecules, NO, and intracellular calcium ions activate cascades that cause ischemic damage (22). MAPKs play an influencing character in this pathological event, whose signaling and associated gene response depend on the severity of IR. Decreased activation of MAPKs can help to overcome the damages attributable to IR (23). Accordingly, the drugs that tone down the activity of MAPKs are beneficial as a therapeutic strategy because they can reduce the synthesis of inflammatory cytokines and their signaling path activity (24).

In 1994, JNK activation was observed in IR-insulted heart and kidney tissues (25). Since this initial discovery, JNK activation has been observed in ischemia-induced impairment in other organs like the liver, intestine, brain, etc. (24). JNK is activated by TNF-α and IL-1, which are the director mediators of IR-induced liver damage. JNK also participates in inflammatory processes by bringing on the expression of dangerous molecules and chemokines (24). TNF-α induced ROS to oxidize and deactivate MAPK phosphatases (MKP), which dephosphorylate and activate JNK for a long time. Long-term activation of JNK due to TNF-α induction requires suppression of NF-κB. In hepatocytes, TNF-α inhibits NF-κB, resulting in long-term activation of JNK and contributes to TNF-induced apoptosis (26). 

After binding TNF-α, its receptor (TNF-R1), a long-term stimulator of JNK, undergoes a structural change, allows them to absorb adapter molecules, and begins activation of the intracellular signaling pathways (27). The intracellular region of TNF-R1 contains an active protein-protein motif with 80 amino acids called the death domain. Antioxidants can prevent long-term JNK activation after TNF-α downstream signaling (28). Therefore, it seems that ROS production triggered by TNF-α can be an impressive factor in long-term JNK activation.

The ROS-JNK pathway, together with TNF-α, induces apoptosis and necrosis in hepatocytes. TNF-α is a potent activator of JNK to phosphorylate c- jun, ATF-2, and JunD (29). These transcription factors are members of the AP-1 family and stimulate the transcription of AP-1-dependent genes, many of which are involved in the regulation of inflammation, proliferation, and cell death (30).

JNK activation begins after the binding of TNF receptor-associated factor 2 (TRAF2) and receptor-interacting protein (RIP) to TNFR1-associated death domain protein (TRADD) that phosphorylates the JNK kinase kinases such as MEK kinase 1 (MEKK1) and apoptosis signal-regulating kinase 1 (ASK1) (31). TRAF2 partially induces JNK activation through a ROS-dependent pathway that activates ASK1. ASK1 plays a role in ROS-induced cell death in a variety of cells. It is involved in the pathogenesis of oxidative stress-related diseases such as neurological and heart diseases, liver disorders due to acetaminophen and alcohol poisoning, and hepatic cancer (32). ASK1 is a serine/threonine kinase belonging to the MAP kinase kinase kinase (MAPKKK) family that activates MAP kinase kinase (MAPKK) and thus activates JNK and p38 MAPK. In eukaryotic cells, this process regulates various cellular responses such as apoptosis, differentiation, cell survival and death, and inflammation. Different stresses, including ROS, TNF α, and lipopolysaccharides activate ASK1 (33).

Suppression of p38 blocks the production and activity of inflammatory cytokines. Research by Karin *et al*. has proved that inhibition of p38 activity prevents the activation of both IL-1α and IL-1β genes. *In vitro*, lipopolysaccharide activates the production of IL-1 in macrophages by stimulating these two genes. In animal models, a p38 inhibitor blocks p38 activation during reperfusion, reduces liver damage, and increases the survival of transplanted tissue (34). Since p38 and JNK signaling pathways overlap in cases such as activation with inflammatory cytokines, possible mechanisms for reducing JNK expression will also apply to p38 (35).

Therefore, suggesting a treatment to regulate the amount and activity of MAPKs will be a profitable strategy in controlling liver IRI, and p38 and JNK inhibitors have high potential therapeutic value. These inhibitors can reduce cell damage, apoptosis, and necrosis resulting from IR (3, 36).

The complexity of the pathological processes of IR has caused no available ideal drug to treat its damages, so it is necessary to provide new and efficient medications to overcome IRI. Medicinal plants have been prevalent since ancient times when chemical medicines were not available (37). Some active ingredients of medicinal plants like riboflavin, green tea polyphenols, ligustrazine, etc., have shown a suitable activity against IR damages, which makes sense to use natural products to alleviate the liver IRI (38-40). Silibinin is a combination of an equal proportion of silybin A & B and is extracted from *Silybum marianum* (milk thistle). It plays a principal role in the development of the biological properties of *Silybum marianum* as the main ingredient of the silymarin (41). According to previous studies, silybin has shown anti-inflammatory effects by inhibiting the expression of cyclooxygenase II, TNF-α, PGE2, and IL-1β (42). Its anti-cancer effects on the lung, prostate, bladder, and thyroid have been demonstrated by stimulating the expression of apoptotic genes such as caspase and Bax (43). 

Our survey showed that silibinin significantly reduces the AST and LDH of the nonspecific index and ALT as the specific index of liver tissue damages, which increased under IR conditions. These enzymes are released due to increased ROS and subsequent peroxidation of the cell and organelle membrane lipids and proteins (44). Researchers have shown that serum levels of these enzymes also are increased as the time of ischemia increases. It seems that silibinin can stabilize the membranes by neutralizing ROS (45).

Because the endogenous antioxidant defense system restrains the production of ROS in the hepatic IR process, enough values of enzymes such as SOD, CAT, and GSH-Px can protect the liver against IRI (46). Besides, the MDA level, as a lipid peroxidation index, can be increased by the overproduction of oxygen free radicals (47). The current study showed that the levels of TAC and GPx were significantly elevated, but the MDA value decreased after the injection of silibinin compared with the IR group. All of these showed that silibinin has notable antioxidant activity in opposition to IR-induced hepatic oxidative damages (48). Pathological studies illustrated that in the SILI + IR group, silibinin reduced vascular endothelium damage, cell degeneration and necrosis, neutrophil infiltration and accumulation, Kupffer cell activation, and cytoplasmic vacuolation (49). 

Silibinin, an anti-oxidant and a ROS scavenger, can neutralize oxygen free radicals and prevent the long-term activation of JNK. It can be one of the possible mechanisms of silibinin in reducing the expression of the p38 and JNK genes (50).

Reem *et al*. showed that a combination of silibinin and vitamin E could prevent p38 activation induced by D-galactosamine/lipopolysaccharide in liver cells by inhibiting ASK1. JNK conducts the cleavage of Bid protein (a pro-apoptotic protein) to form jBid, which causes the release of the second mitochondria-derived activator of caspase (Smac) protein in mitochondria (51). As a result, the activation of JNK in mitochondria in response to ROS causes cytochrome C release and cell death. JNK activation is also associated with hepatic apoptosis (52). The death signaling pathway leading to apoptosis involves the activation of caspase-3 and release of cytochrome C (53). 

Environments with high oxidative stress, such as those shown during reperfusion lead to JNK activation and an increase in ROS. Under IR conditions, JNK activation begins in the mitochondria and requires electron transfer, ROS production, and calcium flux (54). Consequently, modulating JNK activation or reducing its expression by reducing oxygen-free radical production may provide a novel way to prevent IR liver damage. 

Uehara *et al*. showed that the experimental inhibition of JNK with specific selective inhibitors such as CC-401 reduced hepatic apoptosis in mice following liver transplantation by reducing the cytochrome C release and decreasing the caspase-3 activation (55).

Inhibition of JNK significantly reduced non-parenchymal cell death 60 min after reperfusion and peripheral necrosis 8 hr after reperfusion. JNK inhibition reduces liver necrosis and apoptosis, increases connective tissue survival, reduces transplantation injury, and improves liver function after transplantation. JNK inhibition does not affect other signaling pathways, such as p38 and Erk activation (56).

The drug inhibition of JNK activation protects mouse liver cells from apoptosis and promotes cell survival. Pretreatment with JNK inhibitor significantly protects against cell death associated with apoptotic stimuli such as TNF-α and Actinomycin D. This supportive effect is attributed to the expression of anti-apoptotic proteins. Thus, inhibition of JNK suppresses liver damage in the animal model at warm hepatic IR (57).

The above discussion on the role of JNK in hepatocellular IRI highlights the clinical benefits of using JNK expression reducers. JNK reduction may be a new treatment to prevent liver damage after transplantation. Likewise, antioxidants and JNK expression reducers appear to be useful drugs for the treatment of TNF-dependent hepatitis (58). 

According to the results obtained in this study, the antioxidant role of silibinin can be impressive in reducing JNK and inflammatory cytokines expression.

Many p38 downstream targets, once activated, are responsible for activating genes that produce inflammatory cytokines. The primary biological response to p38 activation involves the production and activation of inflammatory mediators to activate leukocytes. Turning on the p38 and p38-dependent pathways in inflammatory processes plays a significant part in post-ischemic liver damage (59).

However, there are restrictions to the use of p38 and JNK inhibitors that require further research. For example, inhibition of p38 increased the proliferation of HSCs, which can contribute to liver fibrosis. Besides, in some other studies, JNK inhibition exacerbated IRI and severe parenchymal degradation. This work was executed using an SP600125 inhibitor of JNK in the mice’s liver injury after IR. The information, although inconsistent with other surveys, confirm the need for further research on the use of JNK inhibitors (21, 60).

## Conclusion

In line with other studies, our results showed that expression of p38 and JNK increases after one hour of ischemia and three hours of reperfusion, and according to the above explanations, ROS production is the most important factor in activating JNK and p38. Overall, silibinin reduces the expression of JNK and p38 genes by neutralizing ROS, thereby altering the expression of inflammatory factors in a way that reduces IRI. This effect diminishes the histological damages caused by the severe inflammatory responses.

## Authors’ Contributions

AK-T and NA conceived the study; AK-T helped with data curation; AK-T and A B provided formal analysis; NA, AB, and MMS did the investigation; AK-T provided supervision; AK-T, AB, and MMS provided validation; AK-T, NMQ helped with writing original draft; AK-T and AB helped with writing, review and editing.

## Funding

This research was funded by the Immunogenetic Research Center (IRC) of Mazandaran University of Medical Sciences, Iran (grant number 1123).

## Institutional review board statement

The study was conducted according to the guidelines of the Declaration of Helsinki, and approved by the Ethics Committee of Mazandaran University of Medical Sciences (IR.MAZUMS.REC.1397.288 and 2018-06-27).

## Informed consent statement

Not applicable.

## Data availability statement

Research data are stored in an institutional repository and will be shared upon request to the corresponding author.

## Conflicts of Interest

The authors declare no conflicts of interest. 
